# 3-[(4-Oxo-4*H*-thio­chromen-3-yl)meth­yl]-4*H*-thio­chromen-4-one

**DOI:** 10.1107/S1600536813001906

**Published:** 2013-02-09

**Authors:** M. Somasundaram, S. Athavan, K. K. Balasubramanian, R. Saiganesh, S. Kabilan

**Affiliations:** a Department of Chemistry, Annamalai University, Annamalai Nagar, Chidambaram, India; b Shasun Reaearch Centre, 27 Vandaloor Kelambakkam Road, Keezhakottaiyur, Meelakottaiyur Post, Chennai, India

## Abstract

The title mol­ecule, C_19_H_12_S_2_O_2_, lies on a twofold rotation axis. The thio­chromonone unit is essentially planar, with a maximum deviation of 0.0491 (14) Å. The dihedral angle between the thio­chromenone ring systems is 64.48 (4)°. In the crystal, there are weak π–π stacking inter­actions, with a centroid–centroid distance of 3.7147 (9) Å.

## Related literature

For backgound to bis-chromonones, see: Santhosh & Balasubramanian (1991 ▶); Panja *et al.* (2009 ▶). For related structures, see: Ambartsumyan *et al.* (2012 ▶); Nyburg *et al.* (1986 ▶); Li *et al.* (2010 ▶).
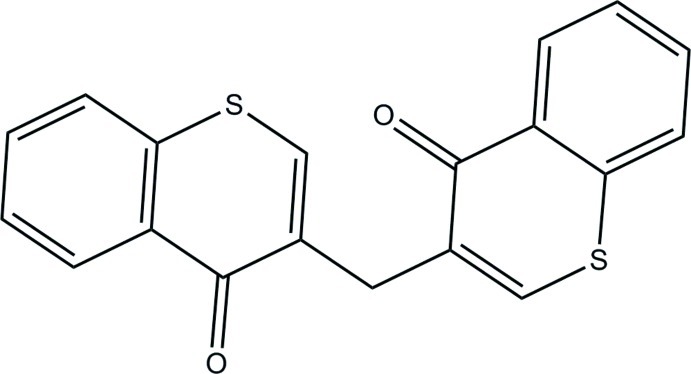


## Experimental

### 

#### Crystal data

C_19_H_12_O_2_S_2_
*M* *_r_* = 336.41
Monoclinic, 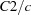

*a* = 11.9480 (5) Å
*b* = 11.8649 (5) Å
*c* = 11.1416 (5) Å
β = 108.918 (2)°
*V* = 1494.14 (11) Å^3^
*Z* = 4
Mo *K*α radiation
μ = 0.36 mm^−1^
*T* = 298 K
0.38 × 0.28 × 0.20 mm

#### Data collection

Bruker SMART CCD diffractometer
Absorption correction: multi-scan (*SADABS*; Bruker, 2007 ▶) *T*_min_ = 0.875, *T*_max_ = 0.931
5040 measured reflections
1631 independent reflections
1410 reflections with *I* > 2σ(*I*)
*R*_int_ = 0.019

#### Refinement

*R*[*F* ^2^ > 2σ(*F* ^2^)] = 0.033
*wR*(*F* ^2^) = 0.118
*S* = 0.88
1631 reflections
109 parameters
H atoms treated by a mixture of independent and constrained refinement
Δρ_max_ = 0.27 e Å^−3^
Δρ_min_ = −0.22 e Å^−3^

### 

Data collection: *SMART* (Bruker, 2007 ▶); cell refinement: *SAINT* (Bruker, 2007 ▶); data reduction: *SAINT*; program(s) used to solve structure: *SHELXS97* (Sheldrick, 2008 ▶); program(s) used to refine structure: *SHELXL97* (Sheldrick, 2008 ▶); molecular graphics: *PLATON* (Spek, 2009 ▶) and *Jmol* (Hanson, 2010 ▶); software used to prepare material for publication: *SHELXTL* (Sheldrick, 2008 ▶).

## Supplementary Material

Crystal structure: contains datablock(s) I, global. DOI: 10.1107/S1600536813001906/lh5551sup1.cif

Structure factors: contains datablock(s) I. DOI: 10.1107/S1600536813001906/lh5551Isup2.hkl

Supplementary material file. DOI: 10.1107/S1600536813001906/lh5551Isup3.cdx

Supplementary material file. DOI: 10.1107/S1600536813001906/lh5551Isup4.cml

Additional supplementary materials:  crystallographic information; 3D view; checkCIF report

**Fig. 2.**
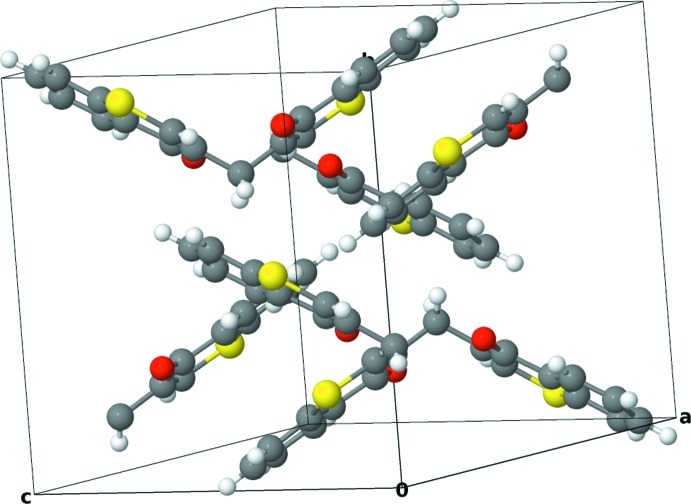
Part of the crystal structure illustrating the π..π stacking interactions.

Enhanced figure: interactive version of Fig. 2

## Figures and Tables

**Fig. 1.**
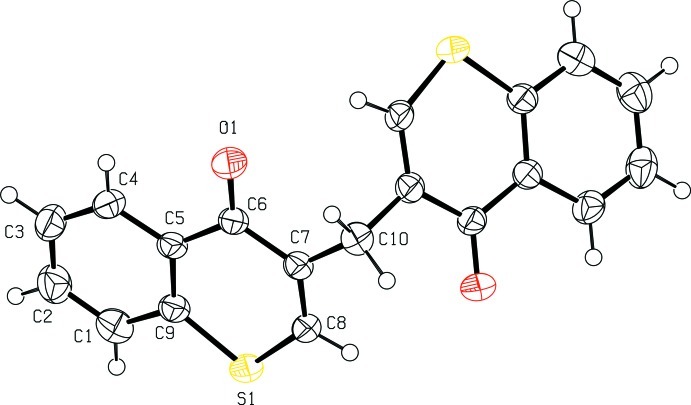
The molecular structure of the title compound, showing 30% probability displacement ellipsoids. Unlabeled atoms are related by the symmetry operator (1-x, y, -z+1/2).

## supplementary crystallographic information

### Comment

Bis-chromonones linked at position 3 are biologically important motifs (Santhosh & Balasubramanian, 1991; Panja, *et al.*, 2009). Analogues of these compounds prepared by replacing the oxygen atom in the heterocyclic core with sulfur are considered to be chemically inportant. Herein, we report the structure determination of the title compound (I).

The molecular structure of (I) is shown in Fig. 1. The molecule lies on a twofold rotation axis. The unique thiochromonone unit is essentially planar with a maximum deviation of 0.0491 (14) Å for atom C6. The planarity of this unit can be attributed to the sp^2^ hybridized nature of the aromatic benzene unit and the fused olefinic thiopyranone unit. This is similar to the case of a methylene bridged chromenone example found in the literature (Ambartsumyan *et al.*, 2012). The dihedral angle between the two thiochromenone ring systems is 64.48 (4)°. The torsion angles about the methylene carbon C10 are 93.05 (13) Å for C8—C7—C10—C7^i^ (symmetry code: (i) -x+1, y, -z+1/2) and -87.80 (11) Å for C6—C7—C10—C7^i^. The angle subtended at the bridging methylene carbon C10 by the olefinic carbons [C7—C10—C7^i^ = 113.66 (17)°] and the olefinic bond length [C7—C8 = 1.344 (2) Å] are close to the respective values in known chromanone systems (Ambartsumyan *et al.*, 2012). Examaples of thiochromone structures already appear in the literature (Nyburg *et al.*, 1986; Li *et al.*, 2010). In the crystal, there are weak π–π stacking interactions (Fig .2) with Cg1···Cg2^ii^ = 3.7147 (9)Å where Cg1 and cg2 are the centroids of the S1/C8/C7/C6/C5/C9 and C1-C5/C9 rings (symmetry code: (ii) 3/2-x, 1/2-y, -z).

### Experimental

To a stirred solution of 4-chloro-2H-thiochromene-3-carbaldehyde (0.5 g, 0.0025 mol) in freshly dried DMSO (6.0 mL) was added dried potassium fluoride (0.3 g, 0.005 mol) and then heated to 343-353K. After completion of the reaction by TLC, the reaction mass was cooled to 303-308K and then quenched with 50 ml of water. The mixture was extracted with ethyl acetate (2 x 30 ml). The combined organic portion was washed with water (2 x 25 mL), dried over anhydrous sodium sulphate and then concentrated under reduced pressure to yield a brown paste. Purification of the crude product by column chromatography yielded the title bis methylene chromanone. 50 mg of the title compound was dissolved in 2 ml of methanol, and warmed to 323K for complete dissolution, then filtered, and the clear solution was stored at room temperature. After 2 days, pale yellow crystals were formed.

### Refinement

H atoms bonded to sp^2^ C atoms were placed in calculated positions with C—H = 0.93Å and U_iso_(H) = 1.2U_eq_(C). The unique H atom conded to C10 was refined independently with an isotropic displacement factor.

### Crystal data

**Table:**

C_19_H_12_O_2_S_2_	*F*(000) = 696
*M**_r_* = 336.41	*D*_x_ = 1.495 Mg m^−^^3^
Monoclinic, *C*2/*c*	Melting point = 489–493 K
Hall symbol: -C 2yc	Mo *K*α radiation, λ = 0.71073 Å
*a* = 11.9480 (5) Å	Cell parameters from 2970 reflections
*b* = 11.8649 (5) Å	θ = 2.5–28.2°
*c* = 11.1416 (5) Å	µ = 0.36 mm^−^^1^
β = 108.918 (2)°	*T* = 298 K
*V* = 1494.14 (11) Å^3^	Block, yellow
*Z* = 4	0.38 × 0.28 × 0.20 mm

### Data collection

**Table:**

Bruker SMART CCD diffractometer	1631 independent reflections
Radiation source: fine-focus sealed tube	1410 reflections with *I* > 2σ(*I*)
Graphite monochromator	*R*_int_ = 0.019
φ and ω scans	θ_max_ = 28.3°, θ_min_ = 2.5°
Absorption correction: multi-scan (*SADABS*; Bruker, 2007)	*h* = −13→13
*T*_min_ = 0.875, *T*_max_ = 0.931	*k* = −15→15
5040 measured reflections	*l* = −8→14

### Refinement

**Table:**

Refinement on *F*^2^	Primary atom site location: structure-invariant direct methods
Least-squares matrix: full	Secondary atom site location: difference Fourier map
*R*[*F*^2^ > 2σ(*F*^2^)] = 0.033	Hydrogen site location: inferred from neighbouring sites
*wR*(*F*^2^) = 0.118	H atoms treated by a mixture of independent and constrained refinement
*S* = 0.88	*w* = 1/[σ^2^(*F*_o_^2^) + (0.1*P*)^2^ + 0.4829*P*] where *P* = (*F*_o_^2^ + 2*F*_c_^2^)/3
1631 reflections	(Δ/σ)_max_ < 0.001
109 parameters	Δρ_max_ = 0.27 e Å^−^^3^
0 restraints	Δρ_min_ = −0.22 e Å^−^^3^

### Special details

**Table:**

Geometry. All e.s.d.'s (except the e.s.d. in the dihedral angle between two l.s. planes) are estimated using the full covariance matrix. The cell e.s.d.'s are taken into account individually in the estimation of e.s.d.'s in distances, angles and torsion angles; correlations between e.s.d.'s in cell parameters are only used when they are defined by crystal symmetry. An approximate (isotropic) treatment of cell e.s.d.'s is used for estimating e.s.d.'s involving l.s. planes.
Refinement. Refinement of *F*^2^ against ALL reflections. The weighted *R*-factor *wR* and goodness of fit *S* are based on *F*^2^, conventional *R*-factors *R* are based on *F*, with *F* set to zero for negative *F*^2^. The threshold expression of *F*^2^ > σ(*F*^2^) is used only for calculating *R*-factors(gt) *etc*. and is not relevant to the choice of reflections for refinement. *R*-factors based on *F*^2^ are statistically about twice as large as those based on *F*, and *R*- factors based on ALL data will be even larger.

### Fractional atomic coordinates and isotropic or equivalent isotropic displacement parameters (Å^2^)

**Table:**

	*x*	*y*	*z*	*U*_iso_*/*U*_eq_	
C1	0.68093 (16)	0.04971 (13)	−0.09101 (16)	0.0468 (4)	
H1	0.6321	0.0231	−0.1689	0.056*	
C2	0.79824 (17)	0.02577 (14)	−0.05263 (17)	0.0510 (4)	
H2	0.8291	−0.0174	−0.1041	0.061*	
C3	0.87278 (15)	0.06556 (14)	0.06363 (17)	0.0481 (4)	
H3	0.9535	0.0509	0.0887	0.058*	
C4	0.82636 (14)	0.12635 (14)	0.14060 (15)	0.0408 (4)	
H4	0.8763	0.1517	0.2186	0.049*	
C5	0.70544 (14)	0.15121 (11)	0.10466 (13)	0.0324 (3)	
C6	0.66133 (13)	0.21467 (12)	0.19414 (13)	0.0340 (3)	
C7	0.53715 (13)	0.24814 (11)	0.15575 (13)	0.0331 (3)	
C8	0.45826 (13)	0.22113 (13)	0.04278 (13)	0.0364 (4)	
H8	0.3814	0.2467	0.0278	0.044*	
C9	0.63250 (13)	0.11413 (12)	−0.01442 (13)	0.0345 (3)	
C10	0.5000	0.31787 (18)	0.2500	0.0384 (5)	
H18	0.4303 (16)	0.3692 (14)	0.2050 (18)	0.043 (5)*	
O1	0.72943 (11)	0.23896 (11)	0.29985 (11)	0.0513 (3)	
S1	0.48288 (3)	0.14521 (3)	−0.07650 (3)	0.04148 (19)	

### Atomic displacement parameters (Å^2^)

**Table:**

	*U*^11^	*U*^22^	*U*^33^	*U*^12^	*U*^13^	*U*^23^
C1	0.0538 (12)	0.0488 (8)	0.0385 (8)	−0.0005 (7)	0.0159 (7)	−0.0020 (6)
C2	0.0548 (12)	0.0519 (9)	0.0530 (9)	0.0107 (8)	0.0265 (8)	0.0016 (7)
C3	0.0365 (10)	0.0540 (9)	0.0568 (10)	0.0096 (7)	0.0190 (8)	0.0131 (8)
C4	0.0316 (10)	0.0500 (8)	0.0374 (8)	0.0021 (6)	0.0066 (7)	0.0101 (6)
C5	0.0314 (9)	0.0372 (7)	0.0278 (7)	−0.0021 (5)	0.0083 (6)	0.0080 (5)
C6	0.0290 (8)	0.0439 (7)	0.0273 (6)	−0.0050 (6)	0.0068 (6)	0.0054 (5)
C7	0.0316 (9)	0.0382 (7)	0.0299 (6)	−0.0017 (6)	0.0103 (6)	0.0062 (5)
C8	0.0269 (9)	0.0492 (8)	0.0319 (7)	0.0002 (6)	0.0079 (6)	0.0060 (5)
C9	0.0340 (9)	0.0386 (7)	0.0297 (7)	−0.0019 (6)	0.0089 (6)	0.0054 (5)
C10	0.0396 (14)	0.0384 (10)	0.0384 (10)	0.000	0.0140 (9)	0.000
O1	0.0351 (7)	0.0820 (8)	0.0311 (6)	−0.0044 (5)	0.0027 (5)	−0.0081 (5)
S1	0.0317 (4)	0.0602 (3)	0.0272 (2)	−0.00364 (15)	0.00220 (19)	−0.00228 (14)

### Geometric parameters (Å, º)

**Table:**

C1—C2	1.356 (3)	C5—C6	1.476 (2)
C1—C9	1.403 (2)	C6—O1	1.2291 (18)
C1—H1	0.9300	C6—C7	1.460 (2)
C2—C3	1.395 (3)	C7—C8	1.344 (2)
C2—H2	0.9300	C7—C10	1.5120 (18)
C3—C4	1.368 (2)	C8—S1	1.7082 (15)
C3—H3	0.9300	C8—H8	0.9300
C4—C5	1.400 (2)	C9—S1	1.7344 (15)
C4—H4	0.9300	C10—C7^i^	1.5120 (18)
C5—C9	1.401 (2)	C10—H18	1.022 (18)
			
C2—C1—C9	120.69 (16)	O1—C6—C5	119.75 (14)
C2—C1—H1	119.7	C7—C6—C5	119.50 (12)
C9—C1—H1	119.7	C8—C7—C6	123.18 (13)
C1—C2—C3	120.35 (16)	C8—C7—C10	120.42 (12)
C1—C2—H2	119.8	C6—C7—C10	116.40 (11)
C3—C2—H2	119.8	C7—C8—S1	127.52 (12)
C4—C3—C2	119.60 (15)	C7—C8—H8	116.2
C4—C3—H3	120.2	S1—C8—H8	116.2
C2—C3—H3	120.2	C5—C9—C1	119.64 (15)
C3—C4—C5	121.49 (15)	C5—C9—S1	123.71 (12)
C3—C4—H4	119.3	C1—C9—S1	116.64 (12)
C5—C4—H4	119.3	C7—C10—C7^i^	113.66 (17)
C4—C5—C9	118.16 (14)	C7—C10—H18	111.2 (10)
C4—C5—C6	118.40 (13)	C7^i^—C10—H18	106.9 (10)
C9—C5—C6	123.43 (14)	C8—S1—C9	102.54 (7)
O1—C6—C7	120.75 (14)		
			
C9—C1—C2—C3	−0.4 (3)	C6—C7—C8—S1	0.0 (2)
C1—C2—C3—C4	1.8 (3)	C10—C7—C8—S1	179.14 (11)
C2—C3—C4—C5	−0.9 (2)	C4—C5—C9—C1	2.8 (2)
C3—C4—C5—C9	−1.4 (2)	C6—C5—C9—C1	−176.99 (12)
C3—C4—C5—C6	178.43 (13)	C4—C5—C9—S1	−176.06 (10)
C4—C5—C6—O1	−3.8 (2)	C6—C5—C9—S1	4.2 (2)
C9—C5—C6—O1	175.94 (13)	C2—C1—C9—C5	−2.0 (2)
C4—C5—C6—C7	175.98 (12)	C2—C1—C9—S1	176.96 (13)
C9—C5—C6—C7	−4.2 (2)	C8—C7—C10—C7^i^	93.05 (13)
O1—C6—C7—C8	−178.10 (14)	C6—C7—C10—C7^i^	−87.80 (11)
C5—C6—C7—C8	2.1 (2)	C7—C8—S1—C9	−0.25 (16)
O1—C6—C7—C10	2.8 (2)	C5—C9—S1—C8	−1.80 (14)
C5—C6—C7—C10	−177.05 (12)	C1—C9—S1—C8	179.31 (11)

Symmetry code: (i) −*x*+1, *y*, −*z*+1/2.
